# A Manual of Medicine

**Published:** 1901-06

**Authors:** 


### Reviews of Books.

**A Manual of Medicine.** Edited by W. H. ALLCHIN, M.D. Vol. I.—*General Diseases*. Pp. x., 442. Vol. II.—*General Diseases* (*continued*). Pp. viii., 380. London: Macmillan and Co., Limited. 1900.

We have here the first instalments of a treatise on medicine which has been planned on thoroughly practical lines. Instead of one or two cumbrous volumes, we have a series of easily-handled little books giving as much matter; instead of a wide range of subjects treated by one man, whose interest, if not his knowledge, must fail at times, we get a series of monographs written by an exceptionally able group of men, each of whom has made a special study of some branch of the whole. There are valuable illustrations and coloured plates, and the recent advances in bacteriology and pathology have been grouped in many cases into special articles, and treated with unusual fulness. We are almost inclined to regret the space taken up by some of these papers in a treatise on practical medicine, but they are excellently done in themselves and the clinical articles are thus relieved from many prolix digressions. It is a great advantage to have full descriptions, for example, of the microscopical technique of blood stains and other tests. And indeed there are a remarkable number of articles of special interest in the book. The chapters on parasites and the diseases caused by them are some of the most fascinating we have seen, and have numerous illustrations. Acute and subacute rheumatism find themselves in a different volume, away from gout, chronic rheumatism, and rheumatoid arthritis; the former are well treated by Dr. Lees, who lays stress on the importance of the disease in childhood, as more frequent, more varied, and more virulent than in adults. Indeed, he finds the age at which the maximum incidence occurs is about ten years. Naturally he lays much stress on the essential character of the heart affection, but we doubt whether there is general agreement as to the very serious view he takes of the rarer, so called, pneumonia in acute rheumatism. We miss any reference to the newer methods of giving the salicylates in difficult cases, such as rectal injections, salophen, and other weaker compounds which are unirritating. A remarkable feature of the work is the number of well-known authorities who have contributed articles—Dr. Luff's able summary of the accepted teaching on gout, Copeman's account of variola, Washbourn, Hale White and Caiger on fevers, are instances. The tropical diseases are discussed in a numerous series of papers, but malaria itself is rather briefly treated, and the only plate of the organisms in the blood which we can find is a solitary figure in the second volume. This is a defect which will, we hope, be remedied in a later edition, for it is hard to overrate the importance of familiarity with the appearance of the protozoa in doubtful cases, even for practitioners in England. In the account of plague, one bacillus is described as the accepted cause of the disease. As a matter of fact the credit is given by many authorities to a totally different organism. Though both are often present, it is very misleading to a student to find the most prominent one quite ignored in the text and plates. Whatever a writer's opinions are, it would not be fair to put forward, say, Friedlander's coccus instead of Fränkel's as the accepted microbe of pneumonia. Probably most readers will turn with interest to the able general papers by Sims Woodhead on "The Infections," by Lazarus Barlow on "Inflammation," by Louis Jenner on "The Blood in Health and Disease," and by Rose Bradford on "The Ductless Glands," which summarise a vast amount of the recent advances in scientific medicine. The difficult subject of the classification of the various forms of meningitis is partly entrusted to G. F. Still, who considers the infective forms under the headings of (1) epidemic, (2) posterior basic, (3) and suppurative meningitis; while the tuberculous, syphilitic, and perhaps some other forms are reserved for treatment elsewhere. It seems somewhat unfortunate that all forms were not considered together for the purposes of comparison, and to prevent the accidental omission of outlying members of the group. However, no classification of disease is without its difficulties, and the general excellence of the work will, we are sure, be endorsed by numerous readers.

